# Immune Suppressive Effect of Cinnamaldehyde Due to Inhibition of Proliferation and Induction of Apoptosis in Immune Cells: Implications in Cancer

**DOI:** 10.1371/journal.pone.0108402

**Published:** 2014-10-01

**Authors:** Franziska Roth-Walter, Anna Moskovskich, Cristina Gomez-Casado, Araceli Diaz-Perales, Kumiko Oida, Josef Singer, Tamar Kinaciyan, Heidemarie C. Fuchs, Erika Jensen-Jarolim

**Affiliations:** 1 Comparative Medicine, Messerli Research Institute, University of Veterinary Medicine Vienna, Medical University of Vienna and University of Vienna, Vienna, Austria; 2 Biotechnology Department, Center for Plant Biotechnology and Genomics, Technical University Madrid, Madrid, Spain; 3 Cooperative Major in Advanced Health Science, Graduate School of Bio-Applications and System Engineering, Tokyo University of Agriculture and Technology, Tokyo, Japan; 4 Comparative Immunology and Oncology, Institute of Pathophysiology and Allergy Research, Center of Pathophysiology, Infectiology and Immunology, Medical University of Vienna, Vienna, Austria; 5 Department of Dermatology, Division of Immunology, Allergy and Infectious Diseases (DIAID), Medical University of Vienna, Vienna, Austria; 6 Department of Biochemical Engineering, University of Applied Sciences Technikum Wien, Vienna, Austria; University of Washington, United States of America

## Abstract

**Background:**

Besides its anti-inflammatory effects, cinnamaldehyde has been reported to have anti-carcinogenic activity. Here, we investigated its impact on immune cells.

**Methods:**

Activation of nuclear factor-κB by cinnamaldehyde (0–10 µg/ml) alone or in combination with lipopolysaccharide was assessed in THP1XBlue human monocytic cell line and in human peripheral blood mononuclear cells (PBMCs). Proliferation and secretion of cytokines (IL10 and TNFα) was determined in primary immune cells and the human cell lines (THP1, Jurkat E6-1 and Raji cell lines) stimulated with cinnamaldehyde alone or in conjunction with lipopolysaccharide. Nitric oxide was determined in mouse RAW264.7 cells. Moreover, different treated PBMCs were stained for CD3, CD20 and AnnexinV.

**Results:**

Low concentrations (up to 1 µg/ml) of cinnamaldehyde resulted in a slight increase in nuclar factor-kB activation, whereas higher concentrations led to a dose-dependent decrease of nuclear factor-kB activation (up to 50%) in lipopolysachharide-stimulated THP1 cells and PBMCs. Accordingly, nitric oxide, interleukin 10 secretion as well as cell proliferation were reduced in lipopolysachharide-stimulated RAW264.7 cells, PBMCs and THP1, Raji and Jurkat-E6 immune cells in the presence of cinnamaldehyde in a concentration-dependent manner. Flow cytometric analysis of PBMCs revealed that CD3+ were more affected than CD20+ cells to apopotosis by cinnamaldehyde.

**Conclusion:**

We attribute the anti-inflammatory properties of cinnamaldehyde to its ability to block nuclear factor-κB activation in immune cells. Treatment with cinnamaldehyde led to inhibition of cell viability, proliferation and induced apoptosis in a dose-dependent manner in primary and immortalized immune cells. Therefore, despite its described anti-carcinogenic property, treatment with cinnamaldehyde in cancer patients might be contraindicated due to its ability to inhibit immune cell activation.

## Introduction

Cinnamon is widely used in the manufacturing industry as a spice and flavoring agent, but it is also an important compound in traditional herbal medicine. The essential oil of the cinnamon bark is constituted by >80% of cinnamaldehyde [1] and the aqueous extract of the cinnamon spice has been attributed with antioxidant properties [2], [3]. Cinnamaldehyde (CA) is a bioactive compound that has been identified to have anti-bacterial [4], [5], anti-inflammatory [6], [7], hypoglycemic [8], anti-mutagenic [9], [10] and anti-tumorigenic activity. Moreover, it was demonstrated to be anti-proliferative and pro-apoptotic on various cancer cell lines *in vitro* [11]–[15]. The anti-carcinogenic property by CA is achieved by mitochondrial depolarization [16] leading to elevated reactive oxygen species, activation of the pro-apoptotic Bcl-2 family proteins, caspase-3 and mitogen-activated protein kinases [16], [17] as well as inhibition of NF-κB and AP-1 activity [15],[18].

Compounds possessing both anti-cancer as well as anti-inflammatory properties like CA may therefore provide an attractive therapeutic tool for cancer therapy. It is well known, that chronic inflammation is a trigger for cancer promotion. However, in an established tumor an immune-suppressive environment already exists and further immune-suppression leads to tumor promotion [19]–[23]. In this respect, death or accumulation of dysfunctional dendritic cells [24], [25], downregulation of HLA class I molecules [26], [27] and increased numbers of regulatory T cells [28] within tumors have gained attention. Immunosuppression in the tumor environment leads to reduced proliferation of peripheral T cells *in vitro*, which correlates with a more negative outcome in cancer patients [29]. Consequently, successful stimulation of the immune system as a result of cancer therapy has been shown to reduce disease relapses and to improve survival [30].

Hence, various strategies in cancer immunotherapy have emerged in recent years to combat immunosuppressive factors and to create an anti-tumor environment. Approaches in active anti-cancer immunotherapy include 1) the introduction of tumor-associated antigens or derivatives thereof as vaccines in an immunogenic context to break tumor tolerance; [31] 2) isolation of immune cells from cancer patients, followed by antigen pulsing and/or stimulation *ex vivo* before re-infusion into the patient [32] as well as 3) blocking immunosuppressive molecules like cytotoxic T lymphocyte-associated antigen 4 (CTLA4), and programmed cell death protein 1 (PD1) with monoclonal antibodies [33].

The anti-tumorigenic properties, which have so far been attributed to cinnamaldehyde, were deduced from models concentrating on cancer cells. However, considering the importance of tumor-infiltrating immune cells, we aimed in this study to critically assess its effects on primary and immortalized immune cells.

## Materials and Methods

### Ethic statement

The study was approved by the institutional ethics committee of the Medical University of Vienna (EK-Nr. 949/2011) and informed written consent was obtained from all subjects before their participation in the study. Healthy volunteers with no reported allergy to cinnamon donated 15 ml blood.

### PBMC isolation and cell lines

Peripheral blood mononuclear cells (PBMCs) of 6 healthy volunteers with no reported allergy to cinnamon were isolated from whole blood using Ficoll-paque density gradient centrifugation as previously described [34], [35].

The THP1-XBlue human monocytic cell line, obtained from InvivoGen (San Diego, CA, USA) as well as THP1, Jurkat E6-1, Raji (all from ATCC, Rockville, MD, USA) cell lines and peripheral blood human mononuclear cells (PBMCs) were maintained in suspension in RPMI-1640 (Gibco Invitrogen, Darmstadt, Germany) containing heat inactivated 10% fetal calf serum (FCS), 1% penicillin/streptomycin and 1% L-glutamine. According to the manufacturer's protocol, 200 µg/ml Zeocin were added upon propagation to THP1-XBlue cells. Murine RAW264.7 macrophages, purchased from ATCC were cultivated in Dulbecco's modified Eagle's medium (DMEM; Gibco Invitrogen, Darmstadt, Germany) supplemented with 10% FCS plus 1% penicillin/streptomycin.

### Cell stimulation

All cells were stimulated with CA (SAFC chemicals supply/Sigma Aldrich, Steinheim, Germany) in a concentration range from 0 up to 10 µg/ml with or without 5 µg/ml (15 000 EU/ml) of LPS from *E. coli* 055:B5 (Sigma, St. Louis, MO, USA).

### Nuclear extraction

PBMCs (1×10^6^ cells/well) were seeded into a 48-well plate and stimulated with CA (0 to 10 µg/ml) alone or in combination with LPS (5 µg/ml, 15 000 EU) for 24 h. Subsequently, nuclear extracts were obtained using a commercial available nuclear extraction reagents and according to manufacturer's protocol (Thermo Scientific, Pierce, Rockford, IL). In brief, cells were washed in PBS and lysed in cytoplasmic extraction reagent I and II. After removing cytoplasmic fraction, nuclear proteins were extracted using nuclear extract reagent and stored at −80°C.

### phospho-NFkB p65 assay

Phospho-NFkB p65 were determined of nuclear fractions using a phospho-NFkB p65 ELISA, (InstantOne ELISA, eBioscience, San Diego, CA) according to the manufacture's protocol. Briefly, to nuclear extracts, an antibody cocktail mix were added for one hour before washing plate and adding detection reagent for 30 minutes, stopping the reaction and measuring optical density at 450 nm.

### NF-κB activation assay

NF-κB activation assays were performed using THP1-XBlue reporter cells, stably expressing an NF-κB/AP-1-inducible secreted alkaline phosphatase reporter (SEAP), according to the company's protocol. In brief, 1×10^5^ cells/well were seeded into a 96-well plate and stimulated with CA (0 to 10 µg/ml) alone or in combination with LPS (5 µg/ml, 15 000 EU/ml) for 24 h. NF-κB activity was determined adding QUANTI-Blue as a substrate of secreted alkaline phosphatase in the supernatants and further incubation for 8 h at 37°C and 5% CO_2_. Subsequently, optical density (OD) was measured at 625 nm using a spectrophotometer Tecan InfiniteM200 PRO (Tecan Group Ltd, Männedorf, Switzerland).

### Nitric oxide determination

Nitric oxide (NO) concentrations of supernatants were determined using Griess reagent (Sigma Chemical Co. St. Louis, MO, USA). RAW264.7 cells (1×10^6^ cells/ml) were incubated with CA (0 to 10 µg/ml) in the presence or absence of LPS (5 µg/ml) for 24 h. Subsequently, equal volume of Griess reagent was added to supernatants. After 10-minute incubation, OD was measured at 550 nm. Zero to 100 µM of sodium nitrite (NaNO_2_; Sigma) was used as standard to calculate NO-levels.

### Cytokine analysis

RAW264.7 cells and human PBMCs (1×10^6^ and 2×10^5^ cells/ml, respectively) were incubated with CA (0–10 µg/ml) in the absence or presence of LPS (5 µg/ml, 15 000 EU/ml). Cell-free supernatants were recovered and stored at −80°C until use. The concentrations of IL10 and TNF-α in the supernatants were determined by ELISA according to manufacturer's protocol (eBioscience, San Diego, CA).

### Cell viability assay

Cell viability was determined with EZ4U (Biomedica, Austria) according to manufacturer's protocol. Two hundreds microliters of the cell suspensions (THP1, 1×10^4^ cells/well; Raji, 2×10^4^ cells/well; Jurkat E6-1, 3×10^4^ cells/well; and human PBMCs, 1×10^5^ cells/well) were incubated in 96-well plates with CA (0–10 µg/ml) in the absence or presence of LPS (5 µg/ml, 15 000 EU/ml) for 24 h. Briefly, 20 µl of substrate solution was added to each well and incubated for 3 h at 37°C, 5% CO_2_, before measuring OD at 465 nm with a reference wavelength of 620 nm.

### Flow cytometric analysis

Cells were stained for Annexin V as a marker for early apoptosis and analysed by FACS. Briefly, freshly isolated human PBMCs (2×10^5^ cells) were incubated with CA (0–10 µg/ml) in the absence or presence of LPS (5 µg/ml, 15 000 EU/ml) for 24 h before staining with anti-CD3-APC (clone SK7) and anti-CD20-PE (clone 2H7) for 30 min at 4°C (both antibodies were from eBioScience and used at a concentration of 1 µl/50 µl staining buffer). Subsequently, AnnexinV–FITC (1 µl/sample, eBioscience, San Diego, CA, USA) in binding buffer (10 mM HEPES, 140 mM NaOH, 2.5 mM CaCl_2_, pH 7.4) were added to cells for 15 min at room temperature. Acquisition and further analysis was performed on FACSCantoII (BD Biosciences).

### Statistical analysis

Statistical analysis was performed using the GraphPad Prism Version 6 software package. Values were analysed using one way analysis of variance (ANOVA) followed by Duncan's multiple range test. P-values of <0.05 were considered significant. All results were expressed as mean ± SD.

## Results

### The effect of cinnamaldehyde on NF-κB/AP-1 activation is concentration-dependent

**Figure 1:**
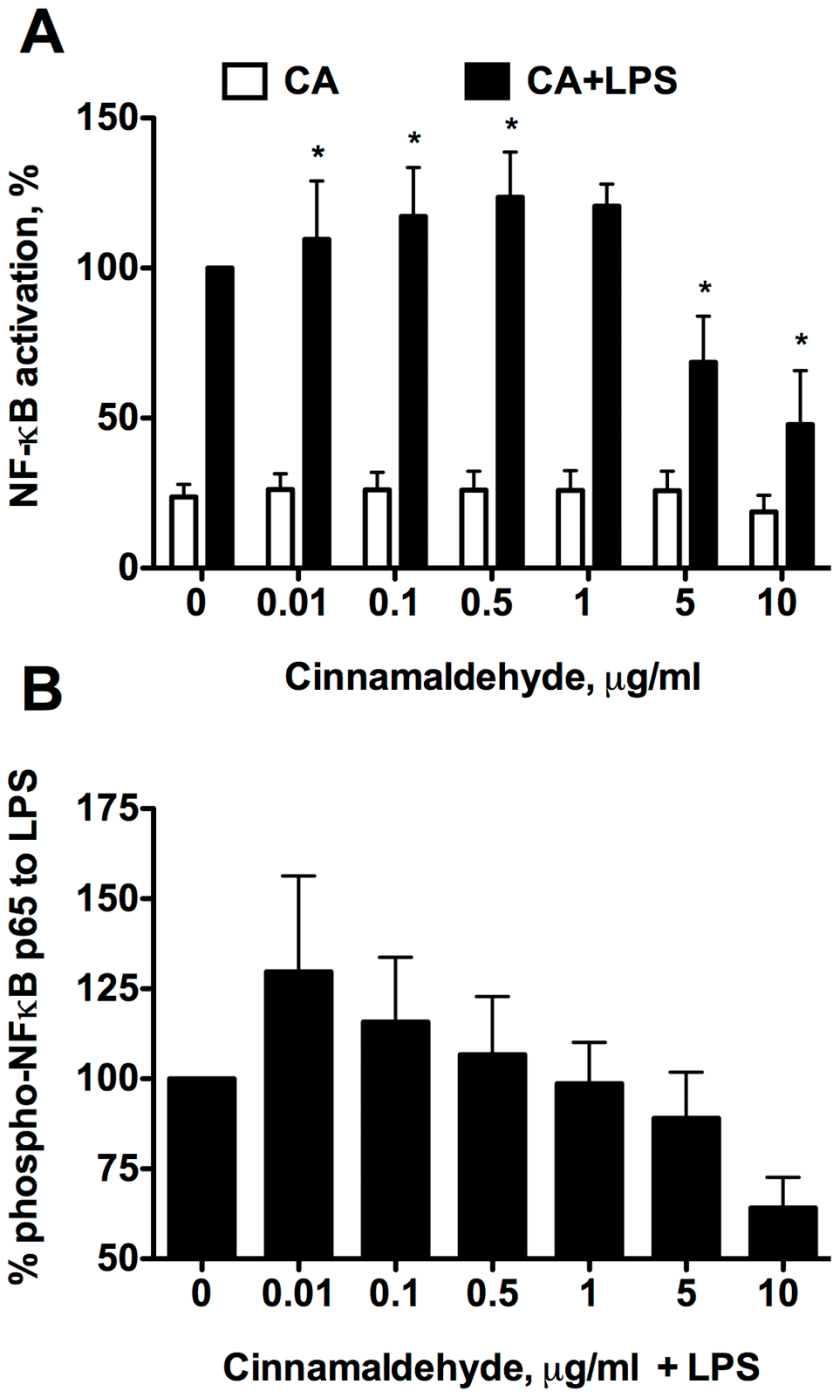
Cinnamaldehyde influences NF-κB activation in human immune cells. A. THP1-XBlue cells or B. PBMCs were treated with different concentrations of CA alone (white bars) or in combination with LPS stimulation (black bars) for 24 h. Bars represent data from A. three independent experiments normalized to LPS alone group and B. three human subjects. Data presented as mean ± SD, * p<0.05.

We first assessed the contribution of cinnamaldehyde on NF-κB activation. CA has been reported to dampen NF-κB activation upon LPS-stimulation, hence acting anti-inflammatory [36], [37]. Similarly, we also observed inhibition of NF-κB/AP-1 up to 65% after LPS-stimulation in THP1-XBlue reporter cells, when concentrations higher than 1 µg/ml were used. However, in contrast to other reports [36], [38]–[40], we stimulated cells also with lower concentrations of cinnamaldehyde and observed a significant increase (up to 40% using 0.1 µg/ml CA) in NF-κB/AP-1 activation when compared to LPS alone (Fig. 1A). Hence, low concentrations of CA further activated NF-κB/AP-1 pathway, whereas higher concentrations inhibited this pathway.

We also assessed NF-κB activation in human immune cells using peripheral blood mononuclear cells (PBMC). Also here very low concentration of CA resulted in further activation of NF-κB as measured by detection of phospho-p65 in the nuclear fractions of LPS-stimulated PBMCs of healthy donor and an increase in the CA-concentration of above 1 µg/ml resulted in hampering NF-κB. Hence, CA was able to inhibit NF-κB activation when using immortalized as well as primary immune cells of human origin in concentration above 1 µg/ml.

### Cinnamaldehyde affects cytokine secretion and nitric oxide in LPS-stimulated immune cells

**Figure 2:**
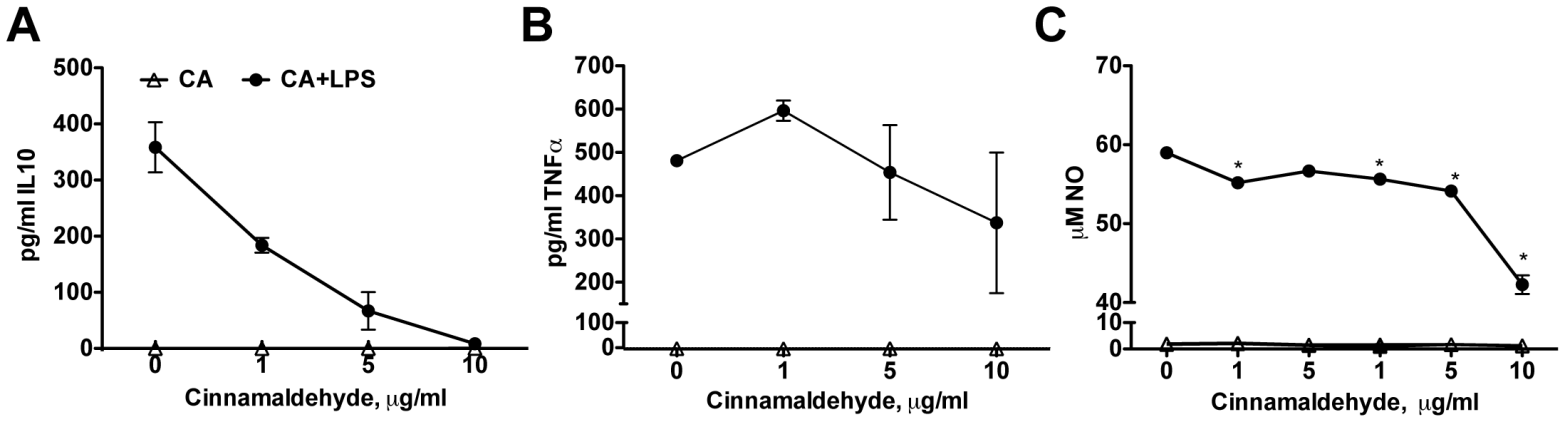
Impact of cinnamaldehyde on cytokine and NO secretion. Human PBMCs were incubated with increasing concentration of CA in the presence (black circles) or absence (open triangle) of LPS for 24 h and supernatants were analysed for A. IL10 and B. TNF-α levels. Data presented as mean ± SD from PBMCs of three subjects. C. Secretion of nitric oxide was determined in RAW264.7, stimulated with increasing concentration of CA in the presence or absence of LPS after 24 h. Representative data from one of three independent experiments, performed in triplicates. Data presented as mean ± SD; * p<0.05, in relation to control samples in each group.

Next we analysed activation of LPS-stimulated immune cells by CA. When LPS-stimulated PBMCs were incubated with CA for cytokine release measurements, concentrations higher than 1 µg/ml inhibited cytokine secretion of immunosuppressive cytokines like IL10 (Fig. 2A) as well as of inflammatory cytokines like TNF-α (Fig. 2B). Concentrations lower than 1 µg/ml did not modify IL10 levels in the supernatants (data not shown). Moreover, no cytokines at all were detected, when cells were incubated with CA alone.

A similar pattern as for the cytokine secretion was oberserved, when analysing NO efflux in LPS-stimulated murine macrophage-like cells (RAW264.7). Also here, NO secretion was impaired (up to 35%) when cells were incubated with higher concentrations of CA (>1 µg/ml). However, no further increase in NO-levels was observed using lower concentrations. NO secretion was not detected in the cells without LPS-stimulation (Fig. 2C).

### Cinnamaldehyde negatively affects viability and proliferation of immune cells

The data so far showed that regardless of cytokines (pro-or anti-inflammatory, TNF-α or IL10), of signaling molecules (NO) as well as pathways (NF-κB/AP-1) investigated, a decrease in activation and production was observed when using higher concentration of CA than 1 µg/ml ( = 8 µM). Therefore, we next tested, whether the observed results where due to reduced viability in CA-treated immune cells.

**Figure 3:**
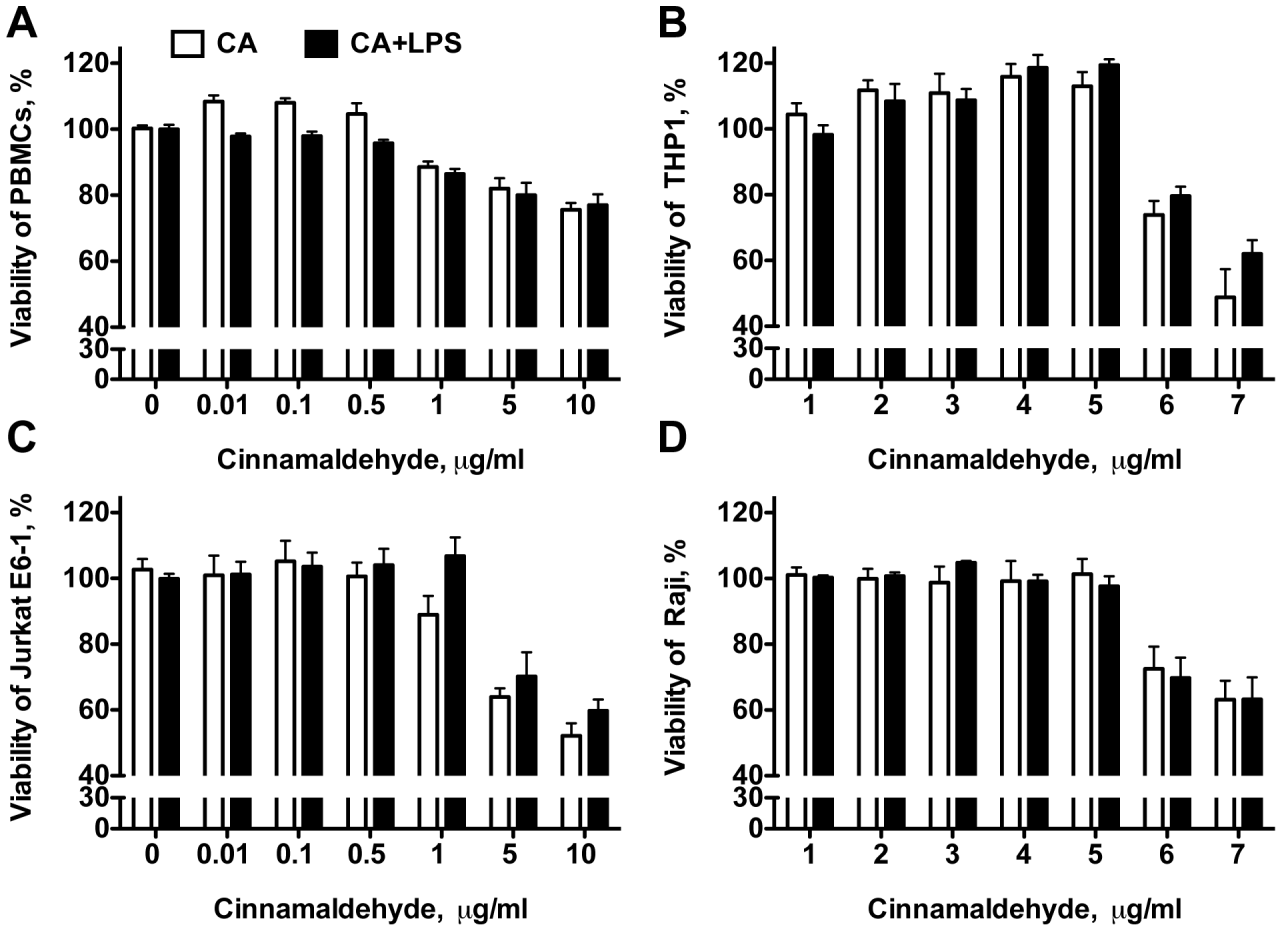
Cinnamaldehyde affects viability and proliferation of human immune cells. A. Human PBMCs, B. THP-1, C. Jurkat E6-1 and D. Raji cells were incubated with increasing concentration of CA in the presence (black bars) or absence (white bars) of LPS. Viable cells were detected using a tetrazolium-based assay. Bars represent data from one of three independent experiments performed in triplicates. Data presented as mean ± SD, * p<0.05, in relation to control samples.

As depicted in Fig. 3, low concentration of CA (<1 µg/ml) led to a significant increase in proliferation of primary human PBMCs, similar to the concentration-dependent NF-κB-profile of CA (Fig. 1). A similar tendency was also observed when using immortalized cells like the human monocyte-like cells THP1, the B cell line Raji and the T cell line Jurkat E6-1, even though this did not reach statistical significance. However, all immune cells tested (THP1, Raji B cells, Jurkat E6-1 and human PBMCs) had in common that higher concentration of CA (>1 µg/ml) significantly reduced cell viability and proliferation. Hence, our data point out that the immune suppressive capacity described by CA is due to its ability to reduce the viabiltiy of immune cells at higher concentrations than 1 µg/ml.

### Cinnamaldehyde rather affects T cells than B cells

**Figure 4:**
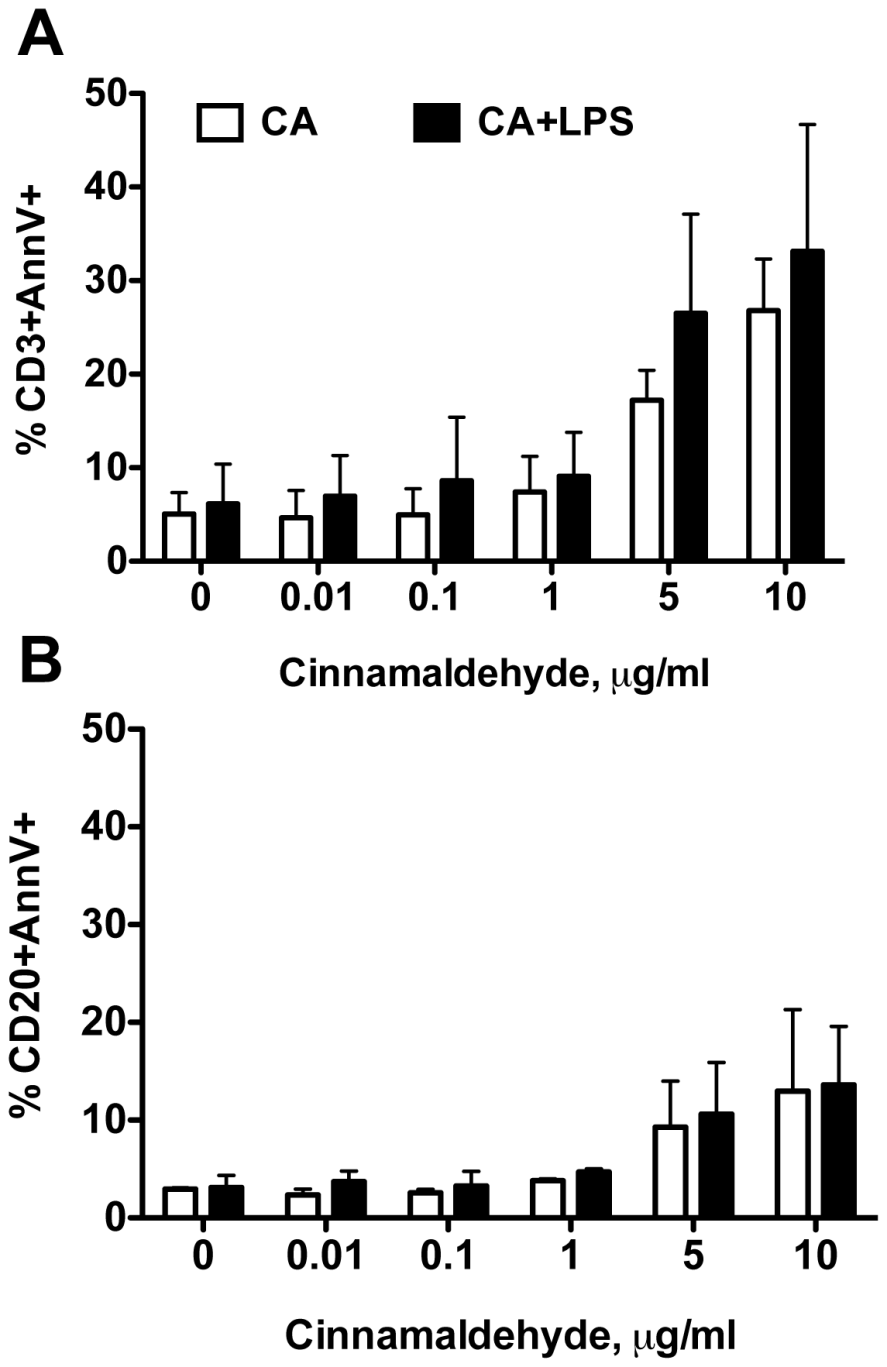
Cinnamaldehyde induced apoptosis in human primary immune cells. Human PBMCs were incubated with CA and stained for CD3, CD20 and AnnexinV and analysed by flow cytometry. Data of three independent experiments are presented as mean ± SD of AnnexinV positive A. CD3 + cells and B. CD20 + cells.

In a next step, we investigated the susceptibility of B and T cells in PBMC-preparations to CA via flow cytometry. PBMCs were stained for CD3 (T cell receptor) as a marker for T cells, CD20 (B lymphocyte antigen) as a marker for B cells and for the early apoptosis marker Annexin V. As depicted in Fig. 4, concentration above 1 µg/ml of CA led to apoptosis of both T cells (Fig. 4A) and B cells (Fig. 4B) in a dose-dependent manner, whereas loading with lower concentrations failed to change cell numbers. Treatment with high concentrations of CA (5 and 10 µg/ml) increased the number of apoptotic T cells up to 8-fold whereas increase of apoptotic B cells was 3.5-fold compared to untreated controls.

## Discussion

CA has been attributed many pharmacological properties, such as being anti-inflammatory, anti-ulcerogenic, antipyretic, antimicrobial, antidiabetic, but also having anti-tumor activity [39]. We intended to investigate these proclaimed prodigious beneficial properties in greater detail, particularly with the focus to understand its impact on immune cells and thereby evaluating its possible indirect contribution to cancer progression. Cancer is defined as the unregulated growth of malignant cells, which is caused in 90–95% by environmental factors such as pollutants, diet, sun exposure, infections, physical inactivity and obesity, and only in 5–10% can be linked to genetic defects [41]. The probability to develop cancer increases with age and has been associated with a decreased immunosurveillance in the elderly [42]. Indeed, the cancer microenvironment is very immunosuppressive and hence reactivation and modulation of the immune system is the primary goal of cancer vaccines [43]. Immune-modulatory regimens offer an attractive approach as they often have fewer side effects than the existing chemotherapeutic drugs. In this respect, blocking of the inhibitory CTLA4 antigen on T cells with anti-CTLA4 antibodies leads to activation of T cells [44], [45] and has been proven to lead to significant survival benefits in two randomized phase III trials in patients with advanced melanoma, emphasizing the importance of immune activation in cancer [46], [47].

Hence, we investigated the immune-modulatory capacity of CA on immortalized and primary immune cells. In our first approach, we investigated its impact on NF-κB activation, since NF-κB is constitutively activated in a number of hematologic and solid tumors and is one of the major transcription factors associated with cancer progression [48], inhibition of apoptosis, tissue invasion and metastasis [49]. Thus, inhibition of NF-κB in malignant cells is regarded beneficial. We were able to reproduce data from literature, that NF-κB activation of LPS-stimulated immortalized monocytes is inhibited by the addition of CA in concentrations above 8 µM [36]–[40], but interestingly we observed a significant increase in NF-κB activation when lower concentrations of CA were used. We thus hypothesize, that an immunosuppressive tumor micro-environment could be shaped when CA could be applied in high enough concentrations. On the other hand low concentration of CA, which is achieved by moderate consumption of cinnamon, further activates the immune system, although not sufficient for combating fast-growing tumors. Hence moderate consumption of CA-containing food seems rather to be a prophylactic, rather than a therapeutical mean. Importantly, our data indicate that high doses might even be contraindicated.

Concerning its bioavailability cinnamaldehyde and its alcohol as well as acid derivate are rapidly absorbed from the gut, metabolized and excreted primarily in the urine, which seems to be independent of dose (up to 250 mg/kg), species and sex. Blood concentrations of cinnamaldehyde following intravenous administration of 5,15, or 25 mg/kg in male and female F344 rats decreased in a biphasic manner [50], [51]. The initial phase correlates with the rapid appearance of cinnamic acid in blood, in which estimated 37–60% of the cinnamaldehyde is oxidized to cinnamic acid within 30 min. The second phase with a half-life of 1.7 h is hypothesized as a release of cinnamaldehyde from protein adducts formed during the initial phase [52]. In a recent study oral administration of CA resulted in a bioavailability of 20% [53]. Moreover in a similar report after oral administration, CA concentration in blood were found to be 1 µg/ml [52] and up to 10 µg/ml for cinnamic acid [54] and maintained for 24 h despite its relatively short biological half-life of 1.7 h.

Hence, high local concentration of cinnamaldehyde and its derivates are achievable *in vivo* upon ingestion and could exert a sustained immune-suppressive effect.

In our next approach, we further focused on the immune-suppressive qualities of cinnamaldehyde by investigating its impact on cytokine secretion in primary PBMCs activated with LPS. In line with suppression of the NF-κB pathway, we observed a concentration-dependent downregulation of the inflammatory and regulatory mediators TNF-α, IL10 and NO being implicated in development and progression of various cancers [55], [56]. However, the prominent decline in mediator secretion of primary immune cells upon incubation with CA suggested that this might be due to changes in the metabolic rate. Indeed, our data clearly demonstrate that CA leads to a pronounced reduction in cell viability of immortalized as well as primary immune cells. Further analysis of the immune cell population in human subjects revealed that especially T cells were more prominently susceptible to CA-induced apoptosis than B cells. The obtained results are highly relevant, since cytotoxic T lymphocytes remain potent mediators of anti-tumor immunity and tumor infiltration by T cells have been shown to be a good prognostic factor in ovarian, colon, breast, renal, prostate and cervical cancers [43]. Furthermore, there are two *in vivo* studies, using cinnamon extract as a cancer therapeutic agent. They report reduced tumor growth of mouse melanoma, when feeding cinnamon extract in large doses (400 µg/g body weight) over 20 to 30 days [37], [57] to mice. However, they also showed a severe reduction of secondary immune organs like the spleen and lymph nodes [28]. In these studies very high doses of CA are used, considering that CA is known to have a low toxicity with a lethal dose low (LDlow) by parental application of 200 µg/g body weight [58]. The observed reduction of fast growing cells like immune and cancer cells might hence be the general characteristic of the described low toxicity of CA.

In contrast, cancerogenic concerns were risen by Mereto et al [59], who proposed cinnamaldehyde as a weak promoter of liver carcinogenesis due to its potential to be clastogenic and its ability to induce micronuclei in rat liver *in vivo*. There exist also one human case report, which associated oral carcinoma formation after the consumption of up to five packs of cinnamon chewing gum a day in a 24-year-old non-smoker [60].

Still, in a 2-year NTP study conducted in mice and rats, in which up to 200 mg/kg body weight of CA, were fed, and which correspond to the maximal concentration of no-observed-adverse-effect-level, NOAEL for long-term effects, no signs of neoplasia were observed [61]. The study highlights the fact, that the consumption of CA may not be cancerogenic when the amount ingested is moderate.

On a molecular level CA as an aldehyde possessing α, β-unsaturated olefinic substituents is known to form adducts with cellular thiol-groups most notably with nonprotein sulfhydryls such as cysteine and glutathione via nucleophilic addition to the β-carbon [62]. Thus, by depriving cells of the antioxidant glutathione, cells are limited in their ability to neutralize free radicals and reactive oxygen compounds. More importantly, depleting cells glutathione-levels also has a severe impact in their iron-metabolism, which ultimately result in the cells' death [63]. Moreover, it is known to act on plasma membrane proteins like the transient receptor potential cation channel subfamily A member 1 (TRPA1) [64], a sensor for environmental irritants, cold and stretch and on TLR4 by preventing their TLR4 oligomerization upon LPS-stimulation, but not by targeting downstream signaling molecules [40]. The consequences of inhibiting TLR4-oligomerization thus are the impairment of NF-κB activation and subsequent secretion of cytokines. In accordance with our data, we show here that not only cancerous cells, but in specific immune cells are highly susceptible to CA leading to inhibition of NF-κB by stabilizing the cell membrane and preventing TLR4 oligomerization as well as inhibiting further activation of the immune cells and the release of cytokines. Moreover, CA induced apoptotosis in lymphocytes, most likely by depriving cells of glutathione.

Since the goal of cancer immune therapy is not to suppress the immune system, but to re-activate resting cells by modulation, which is currently successfully applied when using anti-CTLA4 antibodies, a more differentiated approach for the use of CA has to be advocated for anti-cancer treatment, specifically since it has a pronounced immune suppressive effect on T cells, including the effector T cell population.

Our data indicate that moderate consumption of CA is beneficial for the immune system in the healthy organisms, but that CA-treatment in high doses might be only beneficial when dealing with cancer from hematopoietic cells like lymphoma and leukemia, where the immune system is already suppressed and which account for the most common childhood cancers. However, treatment of other cell-types with CA, to which re-activation of the immune system is highly desired, e.g. carcinomas, sarcomas or germ cell tumors (ovarian or testicular cancer) may contradict its application.

## References

[1] Yeh HF, Luo CY, Lin CY, Cheng SS, Hsu YR, et al (2013) Methods for Thermal Stability Enhancement of Leaf Essential Oils and Their Main Constituents from Indigenous Cinnamon (Cinnamomum osmophloeum). J Agric Food Chem 61: 6293–6298. 23738884 10.1021/jf401536y

[2] Dhuley JN (1999) Anti-oxidant effects of cinnamon (Cinnamomum verum) bark and greater cardamom (Amomum subulatum) seeds in rats fed high fat diet. Indian J Exp Biol 37: 238–242. 10641152

[3] Roussel AM, Hininger I, Benaraba R, Ziegenfuss TN, Anderson RA (2009) Antioxidant effects of a cinnamon extract in people with impaired fasting glucose that are overweight or obese. J Am Coll Nutr 28: 16–21. 19571155 10.1080/07315724.2009.10719756

[4] Ali SM, Khan AA, Ahmed I, Musaddiq M, Ahmed KS, et al.. (2005) Antimicrobial activities of Eugenol and Cinnamaldehyde against the human gastric pathogen Helicobacter pylori. Annals of Clinical Microbiology and Antimicrobials 4. 10.1186/1476-0711-4-20 PMC1373661 16371157

[5] Moleyar V, Narasimham P (1992) Antibacterial activity of essential oil components. International Journal of Food Microbiology 16: 337–342. 1457292 10.1016/0168-1605(92)90035-2

[6] Koh WS, Yoon SY, Kwon BM, Jeong TC, Nam KS, et al (1998) Cinnamaldehyde inhibits lymphocyte proliferation and modulates T-cell differentiation. International Journal of Immunopharmacology 20: 643–660. 9848396 10.1016/s0192-0561(98)00064-2

[7] Reddy AM, Seo JH, Ryu SY, Kim YS, Min KR, et al (2004) Cinnamaldehyde and 2-methoxycinnamaldehyde as NF-κB inhibitors from Cinnamomum cassia. Planta Medica 70: 823–827. 15503352 10.1055/s-2004-827230

[8] Khan A, Safdar M, Khan MMA, Khattak KN, Anderson RA (2003) Cinnamon Improves Glucose and Lipids of People with Type 2 Diabetes. Diabetes Care 26: 3215–3218. 14633804 10.2337/diacare.26.12.3215

[9] De Silva HV, Shankel DM (1987) Effects of the antimutagen cinnamaldehyde on reversion and survival of selected Salmonella tester strains. Mutation Research 187: 11–19. 3540656 10.1016/0165-1218(87)90071-1

[10] Imai T, Yasuhara K, Tamura T, Takizawa T, Ueda M, et al (2002) Inhibitory effects of cinnamaldehyde on 4-(methylnitrosamino)-1-(3-pyridyl)-1-butanone-induced lung carcinogenesis in rasH2 mice. Cancer Letters 175: 9–16. 11734331 10.1016/s0304-3835(01)00706-6

[11] Moon KH, Pack MY (1983) Cytotoxicity of cinnamic aldehyde on leukemia L1210 cells. Drug and Chemical Toxicology 6: 521–535. 6653439 10.3109/01480548309017807

[12] Kwon BM, Lee SH, Choi SU, Park SH, Lee CO, et al (1998) Synthesis and in vitro cytotoxicity of cinnamaldehydes to human solid tumor cells. Archives of Pharmacal Research 21: 147–152. 9875422 10.1007/BF02974019

[13] Jeong HW, Han DC, Son KH, Han MY, Lim JS, et al (2003) Antitumor effect of the cinnamaldehyde derivative CB403 through the arrest of cell cycle progression in the G2/M phase. Biochemical Pharmacology 65: 1343–1350. 12694875 10.1016/s0006-2952(03)00038-8

[14] Koppikar SJ, Choudhari AS, Suryavanshi SA, Kumari S, Chattopadhyay S, et al. (2010) Aqueous Cinnamon Extract (ACE-c) from the bark of Cinnamomum cassia causes apoptosis in human cervical cancer cell line (SiHa) through loss of mitochondrial membrane potential. BMC Cancer 10. 10.1186/1471-2407-10-210 PMC2893107 20482751

[15] Chung WL, Seung HL, Jae WL, Jung OB, So YL, et al (2007) 2-Hydroxycinnamaldehyde inhibits SW620 colon cancer cell growth through AP-1 inactivation. Journal of Pharmacological Sciences 104: 19–28. 17510524 10.1254/jphs.fp0061204

[16] Wu SJ, Ng LT, Lin CC (2005) Cinnamaldehyde-induced apoptosis in human PLC/PRF/5 cells through activation of the proapoptotic Bcl-2 family proteins and MAPK pathway. Life Sciences 77: 938–951. 15964311 10.1016/j.lfs.2005.02.005

[17] Wu SJ, Ng LT (2007) MAPK inhibitors and pifithrin-alpha block cinnamaldehyde-induced apoptosis in human PLC/PRF/5 cells. Food and Chemical Toxicology 45: 2446–2453. 17673346 10.1016/j.fct.2007.05.032

[18] Cabello CM, Bair WB, Lamore SD, Ley S, Bause AS, et al (2009) The cinnamon-derived Michael acceptor cinnamic aldehyde impairs melanoma cell proliferation, invasiveness, and tumor growth. Free Radic Biol Med 46: 220–231. 19000754 10.1016/j.freeradbiomed.2008.10.025 PMC2650023

[19] Condeelis J, Pollard JW (2006) Macrophages: Obligate partners for tumor cell migration, invasion, and metastasis. Cell 124: 263–266. 16439202 10.1016/j.cell.2006.01.007

[20] Mantovani A, Allavena P, Sica A, Balkwill F (2008) Cancer-related inflammation. Nature 454: 436–444. 18650914 10.1038/nature07205

[21] Hanada T, Kobayashi T, Chinen T, Saeki K, Takaki H, et al (2006) IFNγ-dependent, spontaneous development of colorectal carcinomas in SOCS1-deficient mice. Journal of Experimental Medicine 203: 1391–1397. 16717119 10.1084/jem.20060436 PMC2118311

[22] DeNardo DG, Barreto JB, Andreu P, Vasquez L, Tawfik D, et al (2009) CD4 + T Cells Regulate Pulmonary Metastasis of Mammary Carcinomas by Enhancing Protumor Properties of Macrophages. Cancer Cell 16: 91–102. 19647220 10.1016/j.ccr.2009.06.018 PMC2778576

[23] Wang L, Yi T, Kortylewski M, Pardoll DM, Zeng D, et al (2009) IL-17 can promote tumor growth through an IL-6-Stat3 signaling pathway. Journal of Experimental Medicine 206: 1457–1464. 19564351 10.1084/jem.20090207 PMC2715087

[24] Dunn GP, Old LJ, Schreiber RD (2004) The immunobiology of cancer immunosurveillance and immunoediting. Immunity 21: 137–148. 15308095 10.1016/j.immuni.2004.07.017

[25] Gabrilovich DI, Ostrand-Rosenberg S, Bronte V (2012) Coordinated regulation of myeloid cells by tumours. Nat Rev Immunol 12: 253–268. 22437938 10.1038/nri3175 PMC3587148

[26] del Campo AB, Carretero J, Aptsiauri N, Garrido F (2012) Targeting HLA class I expression to increase tumor immunogenicity. Tissue Antigens 79: 147–154. 22309256 10.1111/j.1399-0039.2011.01831.x

[27] Pages F, Kroemer G (2011) Prognostic impact of anticancer immune responses: an introduction. Semin Immunopathol 33: 317–319. 21626162 10.1007/s00281-011-0278-4

[28] Dietl J, Engel JB, Wischhusen J (2007) The role of regulatory T cells in ovarian cancer. Int J Gynecol Cancer 17: 764–770. 17309663 10.1111/j.1525-1438.2006.00861.x

[29] Heimdal JH, Aarstad HJ, Klementsen B, Olofsson J (1999) Peripheral blood mononuclear cell (PBMC) responsiveness in patients with head and neck cancer in relation to tumour stage and prognosis. Acta Otolaryngol 119: 281–284. 10320092 10.1080/00016489950181828

[30] Parkman R, Cohen G, Carter SL, Weinberg KI, Masinsin B, et al (2006) Successful immune reconstitution decreases leukemic relapse and improves survival in recipients of unrelated cord blood transplantation. Biol Blood Marrow Transplant 12: 919–927. 16920557 10.1016/j.bbmt.2006.05.008

[31] Karagiannis P, Singer J, Hunt J, Gan SK, Rudman SM, et al (2009) Characterisation of an engineered trastuzumab IgE antibody and effector cell mechanisms targeting HER2/neu-positive tumour cells. Cancer Immunol Immunother 58: 915–930. 18941743 10.1007/s00262-008-0607-1 PMC3017872

[32] Gupta S, Carballido E, Fishman M (2011) Sipuleucel-T for therapy of asymptomatic or minimally symptomatic, castrate-refractory prostate cancer: an update and perspective among other treatments. Onco Targets Ther 4: 79–96. 21792315 10.2147/OTT.S14107 PMC3143908

[33] Kwek SS, Cha E, Fong L (2012) Unmasking the immune recognition of prostate cancer with CTLA4 blockade. Nat Rev Cancer 12: 289–297. 22378189 10.1038/nrc3223 PMC3433280

[34] Roth-Walter F, Pacios LF, Gomez-Casado C, Hofstetter G, Roth GA, et al (2014) The Major Cow Milk Allergen Bos d 5 Manipulates T-Helper Cells Depending on Its Load with Siderophore-Bound Iron. PLoS One 9: e104803. 25117976 10.1371/journal.pone.0104803 PMC4130594

[35] Roth-Walter F, Gomez-Casado C, Pacios LF, Mothes-Luksch N, Roth GA, et al (2014) Bet v 1 from Birch Pollen Is a Lipocalin-like Protein Acting as Allergen Only When Devoid of Iron by Promoting Th2 Lymphocytes. J Biol Chem 289: 17416–17421. 24798325 10.1074/jbc.M114.567875 PMC4067174

[36] Liao BC, Hsieh CW, Liu YC, Tzeng TT, Sun YW, et al (2008) Cinnamaldehyde inhibits the tumor necrosis factor-alpha-induced expression of cell adhesion molecules in endothelial cells by suppressing NF-kappaB activation: effects upon IkappaB and Nrf2. Toxicol Appl Pharmacol 229: 161–171. 18304597 10.1016/j.taap.2008.01.021

[37] Kwon HK, Hwang JS, So JS, Lee CG, Sahoo A, et al (2010) Cinnamon extract induces tumor cell death through inhibition of NFkappaB and AP1. BMC Cancer 10: 392. 20653974 10.1186/1471-2407-10-392 PMC2920880

[38] Cao H, Graves DJ, Anderson RA (2010) Cinnamon extract regulates glucose transporter and insulin-signaling gene expression in mouse adipocytes. Phytomedicine 17: 1027–1032. 20554184 10.1016/j.phymed.2010.03.023

[39] Liao JC, Deng JS, Chiu CS, Hou WC, Huang SS, et al (2012) Anti-Inflammatory Activities of Cinnamomum cassia Constituents In Vitro and In Vivo. Evid Based Complement Alternat Med 2012: 429320. 22536283 10.1155/2012/429320 PMC3318905

[40] Youn HS, Lee JK, Choi YJ, Saitoh SI, Miyake K, et al (2008) Cinnamaldehyde suppresses toll-like receptor 4 activation mediated through the inhibition of receptor oligomerization. Biochem Pharmacol 75: 494–502. 17920563 10.1016/j.bcp.2007.08.033

[41] Anand P, Kunnumakkara AB, Sundaram C, Harikumar KB, Tharakan ST, et al (2008) Cancer is a preventable disease that requires major lifestyle changes. Pharm Res 25: 2097–2116. 18626751 10.1007/s11095-008-9661-9 PMC2515569

[42] Pawelec G, Derhovanessian E, Larbi A (2010) Immunosenescence and cancer. Crit Rev Oncol Hematol 75: 165–172. 20656212 10.1016/j.critrevonc.2010.06.012

[43] Motz GT, Coukos G (2013) Deciphering and reversing tumor immune suppression. Immunity 39: 61–73. 23890064 10.1016/j.immuni.2013.07.005 PMC3782392

[44] Krummel MF, Allison JP (1996) CTLA-4 engagement inhibits IL-2 accumulation and cell cycle progression upon activation of resting T cells. J Exp Med 183: 2533–2540. 8676074 10.1084/jem.183.6.2533 PMC2192613

[45] Leach DR, Krummel MF, Allison JP (1996) Enhancement of antitumor immunity by CTLA-4 blockade. Science 271: 1734–1736. 8596936 10.1126/science.271.5256.1734

[46] Hodi FS, O'Day SJ, McDermott DF, Weber RW, Sosman JA, et al (2010) Improved survival with ipilimumab in patients with metastatic melanoma. N Engl J Med 363: 711–723. 20525992 10.1056/NEJMoa1003466 PMC3549297

[47] Robert C, Thomas L, Bondarenko I, O'Day S, Weber J, et al (2011) Ipilimumab plus dacarbazine for previously untreated metastatic melanoma. N Engl J Med 364: 2517–2526. 21639810 10.1056/NEJMoa1104621

[48] Oida K, Matsuda A, Jung K, Xia Y, Jang H, et al (2014) Nuclear factor-kB plays a critical role in both intrinsic and acquired resistance against endocrine therapy in human breast cancer cells. Sci Rep 4: 4057. 24531845 10.1038/srep04057 PMC3925966

[49] Naugler WE, Karin M (2008) NF-kappaB and cancer-identifying targets and mechanisms. Curr Opin Genet Dev 18: 19–26. 18440219 10.1016/j.gde.2008.01.020 PMC2587362

[50] Yuan J, Dieter MP, Bucher JR, Jameson CW (1993) Application of microencapsulation for toxicology studies. III. Bioavailability of microencapsulated cinnamaldehyde. Fundam Appl Toxicol 20: 83–87. 8432430 10.1006/faat.1993.1010

[51] Peters MM, Caldwell J (1994) Studies on trans-cinnamaldehyde. 1. The influence of dose size and sex on its disposition in the rat and mouse. Food Chem Toxicol 32: 869–876. 7959441 10.1016/0278-6915(94)90084-1

[52] Yuan JH, Dieter MP, Bucher JR, Jameson CW (1992) Toxicokinetics of cinnamaldehyde in F344 rats. Food Chem Toxicol 30: 997–1004. 1473801 10.1016/0278-6915(92)90109-x

[53] Zhao H, Xie Y, Yang Q, Cao Y, Tu H, et al (2014) Pharmacokinetic study of cinnamaldehyde in rats by GC-MS after oral and intravenous administration. J Pharm Biomed Anal 89: 150–157. 24291110 10.1016/j.jpba.2013.10.044

[54] The Flavor and Fragrance High Production Volume Consortia, consortium TA (2006) Revised Test Plan for Cinnamyl Derivatives. United States Environmental Protection Agency.

[55] Balkwill F (2002) Tumor necrosis factor or tumor promoting factor? Cytokine and Growth Factor Reviews 13: 135–141. 11900989 10.1016/s1359-6101(01)00020-x

[56] Moore KW, De Waal Malefyt R, Coffman RL, O'Garra A (2001) Interleukin-10 and the interleukin-10 receptor. Annual Review of Immunology. pp. 683–765. 10.1146/annurev.immunol.19.1.683 11244051

[57] Kwon HK, Jeon WK, Hwang JS, Lee CG, So JS, et al (2009) Cinnamon extract suppresses tumor progression by modulating angiogenesis and the effector function of CD8 + T cells. Cancer Lett 278: 174–182. 19203831 10.1016/j.canlet.2009.01.015

[58] Kegley SE, Hill BR, Ome S, Choi AH (2010) PAN Pestidice Database. San Francisco, CA: Pesticide Action Network.

[59] Mereto E, Brambilla-Campart G, Ghia M, Martelli A, Brambilla G (1994) Cinnamaldehyde-induced micronuclei in rodent liver. Mutat Res 322: 1–8. 7517500 10.1016/0165-1218(94)90027-2

[60] Westra WH, McMurray JS, Califano J, Flint PW, Corio RL (1998) Squamous cell carcinoma of the tongue associated with cinnamon gum use: a case report. Head Neck 20: 430–433. 9663672 10.1002/(sici)1097-0347(199808)20:5<430::aid-hed12>3.0.co;2-k

[61] (2006) High daily intakes of cinnamon: Health risk cannot be ruled out. Germany: Federal Institute for Risk Assessment, BfR, of the Federal Ministry of Food and Agriculture (BMEL).

[62] Dornish JM, Pettersen EO, Oftebro R (1989) Modifying effect of cinnamaldehyde and cinnamaldehyde derivatives on cell inactivation and cellular uptake of cis-diamminedichloroplatinum(II) in human NHIK 3025 cells. Cancer Res 49: 3917–3921. 2736532

[63] Kumar C, Igbaria A, D'Autreaux B, Planson AG, Junot C, et al (2011) Glutathione revisited: a vital function in iron metabolism and ancillary role in thiol-redox control. EMBO J 30: 2044–2056. 21478822 10.1038/emboj.2011.105 PMC3098478

[64] Olsen RV, Andersen HH, Moller HG, Eskelund PW, Arendt-Nielsen L (2014) Somatosensory and vasomotor manifestations of individual and combined stimulation of TRPM8 and TRPA1 using topical L-menthol and trans-cinnamaldehyde in healthy volunteers. Eur J Pain. 10.1002/j.1532-2149.2014.494.x 24664788

